# Experimental and Constitutive Modelling of Viscoelastic Responses in Carbon Black-Filled Natural Rubber Under Cyclic and Relaxation Loading

**DOI:** 10.3390/polym17233091

**Published:** 2025-11-21

**Authors:** Savaş Kayacı, Necmettin Kaya

**Affiliations:** 1 KE Engineering Co., Ltd., Bursa 16285, Turkey; 2 Department of Mechanical Engineering, Faculty of Engineering, Bursa Uludag University, Bursa 16059, Turkey; necmi@uludag.edu.tr

**Keywords:** natural rubber, viscoelasticity, constitutive modelling, hysteresis, stress relaxation, Bergström-Boyce model

## Abstract

Elastomeric materials exhibit complex time-dependent behaviour under mechanical loading, necessitating accurate constitutive models for industrial applications. This study investigates the hyperelastic and viscoelastic responses of two carbon black-filled natural rubber compounds (50 ShA and 60 ShA) through cyclic shear/compression tests and stress relaxation experiments. The Arruda–Boyce model captures equilibrium behaviour, while the Bergström–Boyce model predicts transient viscoelasticity without relying on Prony series. Considering the results obtained it can be concluded that quantitative hysteresis analysis shows 7–26% energy dissipation, dependent on hardness and strain rate. Relaxation rates (10^−6^–10^−7^ s^−1^) inversely correlated with hysteresis, validated by FEM simulations. A deviation of <3.5% between experiments and simulations confirms the model’s robustness for long-term viscoelastic predictions. This framework enables the efficient design of rubber components (e.g., seismic isolators, seals) requiring prolonged durability under load.

## 1. Introduction

Elastomeric materials are used in almost every mechanical system in every field of industry. The tyre industry uses most of the elastomer supply worldwide. Various polymeric materials are used to manufacture a tyre, such as natural rubber (NR), butadiene rubber (BR), styrene butadiene rubber (SBR), and halogenated polyisobutylene rubber (XIIR) [1]. The transient and frequency-dependent responses of these polymers fundamentally define critical performance metrics like traction, stability, abrasion resistance, and durability [2]. Natural rubber is also a major material used in anti-vibration and shock mounts. In the automotive industry, engine mounts, axles, and suspension bushes are vital components to define any vehicle’s overall ride and handling properties [3]. Similarly, in the railway industry, vehicles have various suspension components made of rubber materials to enhance both stability and comfort of the ride [4]. All these components have both hyperelastic and viscoelastic properties changing with temperature, time, frequency, amplitude and preload. Therefore, engineers and scientists are continuously developing new mathematical models and methods to predict static and transient mechanical behaviour of elastomeric materials, which exhibit very large deformations without breaking and damping due to the hysteretic deformation of their long molecular chains. The mechanical properties of elastomeric materials depend on many factors like ambient temperature, humidity, exposure to sunlight, frequency, amplitude of loads being exerted, etc. To obtain a good and reliable mathematical viscoelastic and hyperelastic material model for elastomers, various tests need to be carried out to have the corresponding stress and strain relationships (stress–strain data couples). These data are then used to find the best coefficients and constants that best fit the experimental data. The tests can be listed as uniaxial compression, uniaxial tension, simple shear, pure shear, volumetric compression, and biaxial tension. This study used uniaxial compression and simple shear tests to obtain the necessary material models.

One of the foundational studies performed was published by Rivlin [5] to introduce the strain energy functions to model the hysteresis of elastomer-like materials. To better understand the time-dependent material response of elastomers, Gross introduced a cornerstone study for modelling viscoelastic behaviour including hysteresis, using linear viscoelastic theory [6]. To predict the responses of elastomer-like materials under large deformation, Gent presented a very simple and effective constitutive model [7]. Arruda and Boyce introduced the 8-chain model, which is widely used to describe the hyperelastic and hysteretic behaviour of elastomers under large deformations [8]. Bergström and Boyce came up with a theory to describe the time-dependent behaviour of elastomers using equilibrium and time-dependent networks [9]. Another mathematical model was introduced to describe hysteresis in elastomers under cyclic loadings using energy dissipation and strain-rate effects [10]. In another classic work, a constitutive model was introduced to capture both hysteresis and creep in elastomeric materials [11]. A different approach was introduced as a thermodynamic framework for modelling hysteresis in elastomers using internal variables and energy dissipation [12]. Some studies introduced combined models to predict the Mullins effect and hysteresis under cyclic loading [13]. Another important factor to predict the viscoelastic behaviour of elastomers is the value of strain rate. A new approach was introduced to describe the mechanical behaviour of an elastomer under various strain-rate conditions [14]. A well-known study was performed by Qui et al. [15] to introduce a non-linear viscoelastic model to capture hysteresis in elastomers, focusing on energy dissipation and strain-rate dependence. An extensive comparison was made by [16] between linear and non-linear material models to describe the viscoelastic behaviour of elastomeric materials. There are also experimental works that were conducted to investigate the influence of elastomer compound ingredients on creep and relaxation behaviour. Such work was performed by Mostafa et al. [17] using SBR and NBR compounds of different formulations. Recent studies made further contributions to the above methodologies to increase computational methods’ reliability. A work has been conducted to investigate the relationship between hysteresis, relaxation, and self-heating during cyclic loadings for elastomers by Luo et al. [18]. Merckel et al. introduced a new model that connects Mullin’s effect, hysteresis, and creep for filled rubber materials [19]. Park et al. discussed hysteresis and creep, which take place during non-linear loading simultaneously. They demonstrated that hysteretic energy dissipation under large deformations must be accounted for to create accurate long-term creep predictions [20]. One study by Wrana and Eberlein introduced a material model for filled vulcanizates and showed the justification of their model using direct comparisons of physical test data to the data obtained in analysis results [21].

Despite these advancements, certain challenges and limitations persist in the practical application of the models explained in this introduction for long-term performance prediction. A primary challenge lies in the accurate characterization of long-term viscoelastic phenomena, such as stress relaxation, using models that are both physically meaningful and computationally efficient. The widely used Prony series approach [22], while effective, requires direct fitting to relaxation or creep data, which can be time-consuming to obtain. The Bergström–Boyce model offers a compelling alternative, as it derives its viscoelastic parameters from the rate-dependence observed in cyclic hysteresis tests. However, a clear research gap exists in the comprehensive validation of this approach for predicting long-term relaxation behaviour solely from short-term cyclic data, particularly for carbon black-filled natural rubber across different hardness grades. Many studies calibrate models against the same type of test they aim to predict, but the robust prediction of long-term relaxation from independent, short-term cyclic tests is a non-trivial task that requires further demonstration. The question of how reliably the model parameters, identified from hysteresis loops, can extrapolate behaviour to prolonged static loading remains a pertinent issue for engineers designing components like seismic isolators or long-life seals.

This study aims to address this gap by presenting a validated framework for predicting the long-term stress relaxation of carbon black-filled natural rubber using a constitutive model identified exclusively from cyclic tests. One of the primary novelties and contributions of this work can be stated to demonstrate that the Bergström–Boyce model, calibrated solely against hysteresis data from two strain rates, can accurately predict independent stress relaxation tests over 3600 s, without relying on Prony series fits from relaxation data. Providing a fully detailed methodology—from specimen curing and experimental testing to finite element implementation—for characterizing two NR compounds (50 and 60 ShA) and validating the model’s predictions for both hysteresis energy loss and normalized stress decay is another novelty of this work. Another novel approach that this study brings forward is to quantitatively show that this approach yields a reliable deviation between experiments and simulations for long-term relaxation, establishing its robustness for engineering applications requiring prolonged durability under static load.

To briefly underline the procedure followed in this paper; quad-pad simple shear specimens and disc specimens of two different carbon black-filled natural rubber materials of 50 ShA and 60 ShA vulcanized and tested under cyclic loadings with two different strain-rate values in shear and compression. The same specimens also underwent relaxation tests at 20% and 30% strain values for 3600 s while recording the change in reaction force. The results were used to determine hyperelastic and viscoelastic material constants and coefficients using Aruda–Boyce and Bergström–Boyce constitutive models. The resulting material models were used to predict the relaxation behaviour of specimens without using the Prony series approach [22]. The results of experiments and computations were explicitly compared and the efficiency of Bergström–Boyce’s model [9] was discussed. In real engineering problems, this approach can be used to predict the viscoelastic life of components working under relatively higher static preloads and to predict the long-life performance of sealing components confined in an enclosure.

## 2. Materials and Methods

**Figure 1:**
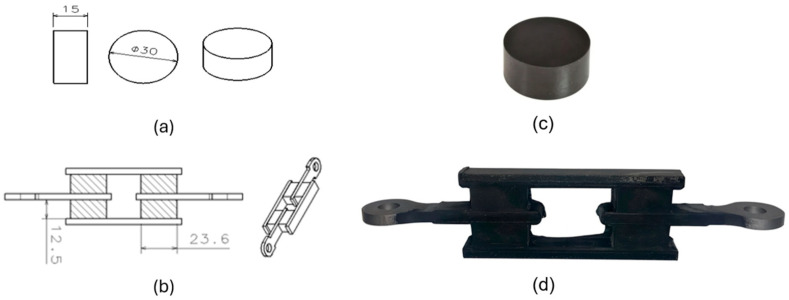
Geometry of samples: (**a**) Compression. (**b**) Simple shear. (**c**) Vulcanized compression sample. (**d**) Vulcanized simple shear sample.

The materials used to cure samples were mixed using two different ratios of natural rubber and carbon black to obtain two nominal hardness values: 50 ShA and 60 ShA. Together with other ingredients like curing agents, anti-ageing agents, mineral oil, etc., the resulting uncured mixture is called a compound. Material with a nominal hardness of 50 ShA has a compound formulation of SVR CV60: 100 phr (sourced from Phu Rieu Rubber Co. Ltd., Phu Rieng District, Vietnam), FEF N550: 31.5 phr (sourced from PCBL Chemical, West Bengal, India), Paraffinic oil: 6.8 phr (sourced from Petroyag A.Ş., Kocaeli, Türkiye), Zinc Oxide: 6.0 phr (sourced from Hepşen Co. Ltd., Istanbul, Türkiye), Stearic acid: 2 phr (sourced from Permata Group, North Sumatra, Indonesia), IPPD: 3 phr (sourced from Shandong sunsine chemical Co., Ltd., Shandong, China), TMQ: 2 phr (sourced from SI Group-Switzerland GmbH, Pratteln, Switzerland), ActiplastT: 2 phr (sourced from Eigenmann & Veronelli S.p.A., Rho (MI), Italy), TBZTD: 0.5 phr (sourced from MLPC International, Rion des Landes, France), TBBS: 0.8 phr (sourced from MLPC International, Rion des Landes, France), S80: 2.54 phr (sourced from Vibiplast Srl., Costano Prima (MI), Italy), and it is referred to hereafter as NR50. Material with a nominal hardness of 60 ShA has a compound formulation of SVR CV60: 100 phr, FEF N550: 62.5 phr, Paraffinic oil: 3.8 phr, Zinc Oxide: 6.2 phr, Stearic acid: 2 phr, IPPD: 3 phr, TMQ: 2 phr, ActiplastT: 2 phr, TBZTD: 0.5 phr, TBBS: 0.8 phr, S80: 2.08 phr, and it is referred to hereafter as NR60. Two mould tools were designed and manufactured to cure the samples using the transfer vulcanization technique. The geometries of the samples are given in Figure 1.

Compression disc and simple shear samples are vulcanized (cured) using a conventional vulcanization press with a clamping capacity of 250 tons. A vulcanization press is a hydraulic press equipped with a main cylinder and two hot platens. One of the hot platens is pressed by the main cylinder against the other while having the mould tool between them. The hot platens are equipped with electric resistances which continuously generates heat fluxing towards the cavity of the mould tool. The curing process is completed under pressure while the tool is being heat fluxed by the electric resistances of the hot platens. The sample shown in Figure 1c does not need any metal insert and it is made of pure rubber raw material. On the other hand, the sample shown in Figure 1d needs 4 metal inserts (made of stw24 steel raw material) put in the tool cavity prior to the transfer of uncured compound into the mould cavity. These metal inserts are shown in Figure 1b without hatching. In the same figure, the rubber blocks are shown by hatched rectangles. To have a perfect bonding between the rubber and steel inserts, steel inserts are two layers of bonding agents applied before they are placed into the cavity of mould tool. The bonding agents used are Chemosil 211 (primary layer) and Chemosil 411 NL (secondary layer). These bonding agents are products of Henkel Corporation, Rocky Hill, CT, USA and widely used in the rubber–metal industry.

The samples are cured using the parameters given in Table 1. The temperature values were determined using the results obtained from rheometer tests for each compound. The objective was to select the temperature value at which the ratio of lower modulus to higher modulus is maximized. Due to the lack of proper equipment (e.g., ALPA Tech. RPA2000 Rubber Process Analyser), it was not possible to measure the cure intensity of components under different vulcanization parameters; instead, components were cured using constant mould temperature and pressure while changing the curing duration. The optimum durations are selected to give the maximum value of stiffness in the quasi-static test and the minimum value of hysteresis. To save the novelty of this technique, further technical details about determining optimum vulcanization parameters for a component from any given shape is not given in this study.

After successfully curing samples using the corresponding tools without any physical defects, every sample was dimensionally measured and recorded. The measurement results are given in Table 2 and Table 3 for compression and simple shear samples, respectively. At least 4 measurements for each geometrical property and average values were taken to calculate corresponding stress and strain values.

The measurement results of samples given in Table 2 and Table 3 are used to calculate corresponding stress–strain values from the load and displacement values obtained in the quasi-static tests carried out in two strain-rate values. The tests were performed using a Zwick Z100 test machine (Manufactured by Zwickroell in Ulm, Germany). To carry out the quasi-static tests, the test parameters given in Table 4 and Table 5 were used for compression and simple shear samples, respectively. During these tests, the application of a preload is important because it helps to reduce the contact problems at the test fixture interfaces. Namely, the preload value of 10 N is necessary to ensure that the surfaces of test fixtures and samples are in full contact with each other without any gap. Preconditioning cycles are needed to eliminate the Mullin’s effect [23] to obtain the load displacement relationships as steadily as possible. The number of test cycles is 4 in each test: the first three were used to eliminate Mullin’s effect and the last one was used to collect the stress–strain data. To have the data at a lower strain rate, the test speed of the Zwick Z100 machine was selected as 20 mm/min. To have the data at a higher strain rate, the test speed of the Zwick test machine was selected as 50 mm/min. The corresponding strain values are calculated using these test speeds for each test sample type (simple shear and uniaxial compression). These strain rates chosen to be within the scope of this study were determined considering the limits of the test machine used. One can select boundary conditions for different engineering problems in higher or lower strain-rate values.

**Figure 2:**
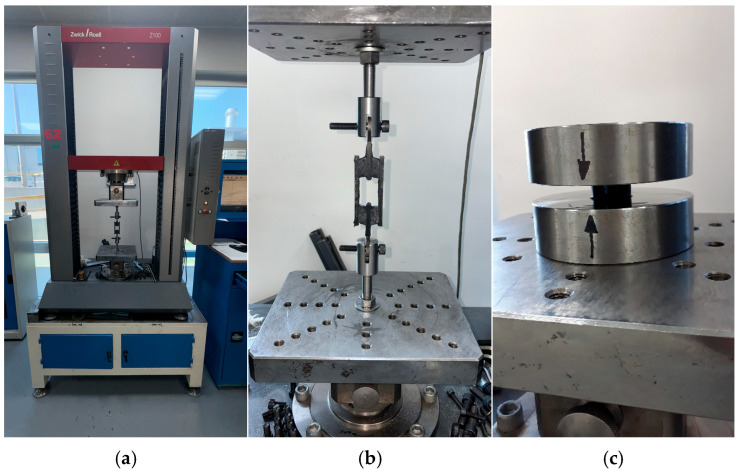
Test setups (**a**) Overall test setup; (**b**) Close-up shear test setup; (**c**) Close-up compression test setup.

To test the simple shear samples, samples are fixed to the test setup through the holes present in the metal inserts at the upper and lower ends of the samples using two bolts as shown in Figure 2b. To apply displacements needed to create cyclic and relaxational loads, the upper metal insert is moved upwards and downwards with a specified strain rate while keeping the lower metal insert fixed stationary.

To test the samples under compression, the compression samples are put between two rigid steel plates as shown in Figure 2c. To apply displacements needed to create cyclic and relaxational loads, the upper steel plate is moved downwards and upwards by the cross of the test machine while keeping the lower rigid steel plate fixed stationary. To eliminate the bulking of compression samples, upper and lower surfaces of samples are lightly oiled to lower the friction as much as possible.

During the cyclic quasi-static tests and relaxation tests, displacement (d) and load (F) values are recorded by the test device. For the loads applied under compression, the true strain (ε) can be calculated by using natural logarithmic ratio of *d* to the height of each sample. The true stress (σ) can be calculated by dividing the load (F) value to the cross-sectional area, which is perpendicular to the direction of load being applied. This area is not constant and changing as the compressive strain decreases. For the loads applied under shear, shear strain (ϑ) can be calculated by dividing the half the value of displacement (d) to the value of height of each rubber block shown in Figure 1b. The shear stress (τ) can be calculated by dividing half the load $\left(F\right)$ value by the cross-sectional area of one rubber block, which is parallel to the direction of load being applied.

Test limits were chosen considering the geometrical limits of samples during each mode of deformation (compression and shear). To minimize the shape factor, which increases the effective compressive modulus of the compression sample, a nominal maximum strain value of ε=−0.5 mm/mm was selected to ensure the measured load values reflect only the material not the geometry. The maximum test limit of ϑ=1 mm/mm for simple shear samples was chosen to lower the bending moment contributions while the rubber pads were loaded in shear [24]. The Bergström–Boyce model needs stress–strain data at two different strain-rate levels (high and low), and this is why all quasi-static tests were performed at two different strain rates [9].

After the completion of quasi-static cyclic tests, the load–displacement and corresponding stress–strain data can be obtained at each cycle at each applied strain rate. The values collected at the latest cycle (4th cycle) are considered to be the equilibrium mechanical properties of each material at each value of strain rate under compressive and shearing loads. In this study, the constitutive model coefficients are obtained using the stress–strain data pairs obtained in the last cycle (4th cycle) of quasi-static tests.

After completing the quasi-static tests, for each sample, a relaxation test was carried out at constant strain levels of ϑ=0.2 and ϑ=0.3 in shear mode for t=3600 s. Similarly, relaxation tests were carried out at constant strain levels of ε=−0.2 and ε=−0.3 in compression mode for t=3600 seconds. These values were chosen because, in many engineering applications, rubber components are designed to work under a mean strain value of maximum 0.3 (i.e.,ε=−0.3,ϑ=0.3) under normal conditions. During relaxation tests, the load (F) value is recorded against time (t) for each mean strain level under each loading mode. Then the value of load at any time is divided by the initial value of load at each strain level to find the normalized load value against time. The value of normalized load gives a better understanding of change in viscoelastic mechanical properties of materials against time.

**Figure 3:**
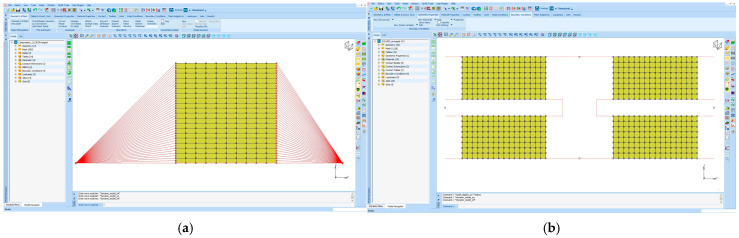
FEM definitions using Marc 2024.2 software. (**a**) Axi-symmetrical 2D compression problem. (**b**) Plain strain 2D shear problem.

The same problems with the same initial and boundary conditions explained up to here are also modelled and described using an FEM software called Marc 2024.2 (a trademark of Hexagon Company) as shown in Figure 3.

## 3. Experimental Test Results

The samples, whose dimensions are given in Table 2 and Table 3, were tested according to the test parameters given in Table 4 and Table 5. The results are illustrated in Figure 4, Figure 5, Figure 6, Figure 7, Figure 8, Figure 9, Figure 10 and Figure 11.

There are obvious differences in the form of loading and unloading curves for every test carried in compression and shear. Clearly the area between the loading and unloading curves is much higher for compressive tests than shear tests. This is a clear sign that under compressive loads the internal friction between elastomer molecules are higher compared to the friction exhibited under shearing loads. Having the same tests at two strain-rate values slightly increased the reaction loads measured at the same displacements, thus, the stress values measured were also slightly higher at the same strain levels when using higher strain-rate values. Another observation is that as the hardness increases, the area between the loading and unloading curves increases. These differences in the properties of the NR50 and NR60 samples do not give us an absolute reasoning to decide which compound is better compared to the other. The users of these materials can select the best one that fits in the favour of the features of the component to be manufactured. For example, if there is a necessity to absorb a shock caused by a specific excitation source, the NR50 compound can fit better because it has higher potential to absorb more energy compared to the NR60 compound. Or, if there is a relatively higher weight to be suspended, NR60 can be a better candidate because of its higher modulus value compared to the NR50 compound. The reason why two materials of different rubber formulations were studied in this work is only to justify the methodology using two different rubber raw materials.

For every test carried out at different strain rates, load and displacement pairs were grouped in two domains: loading and unloading. The loading group represents the load–displacement data pairs while the value of applied load increases, while the unloading group represents the data pairs while the value of applied load decreases.

**Figure 4:**
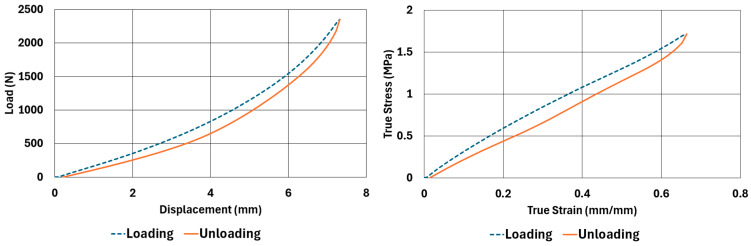
NR50 sample—compression test results at 0.0553 1/s strain rate.

**Figure 5:**
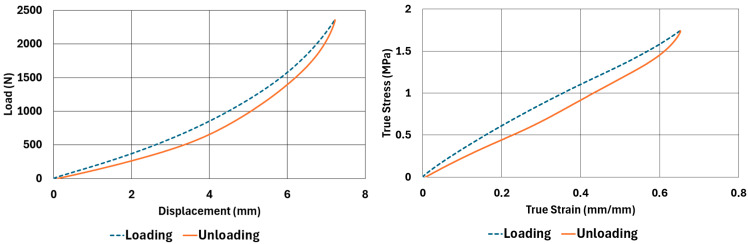
NR50 sample—compression test results at 0.0221 1/s strain rate.

**Figure 6:**
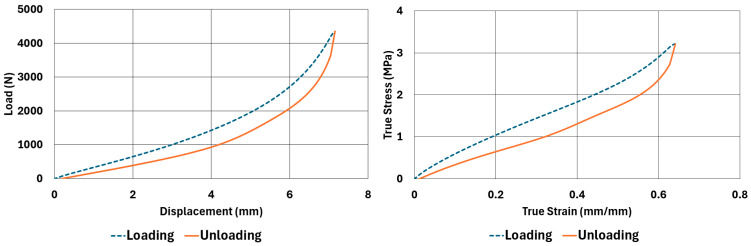
NR60 sample—compression test results at 0.0553 1/s strain rate.

**Figure 7:**
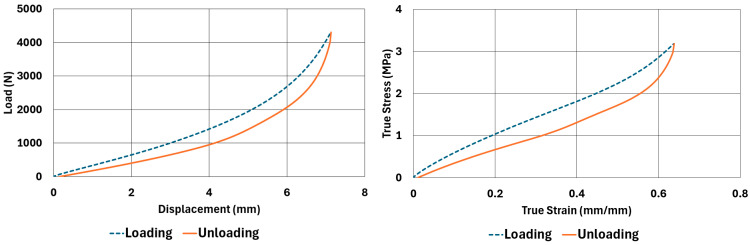
NR60 sample—compression test results at 0.0221 1/s strain rate.

**Figure 8:**
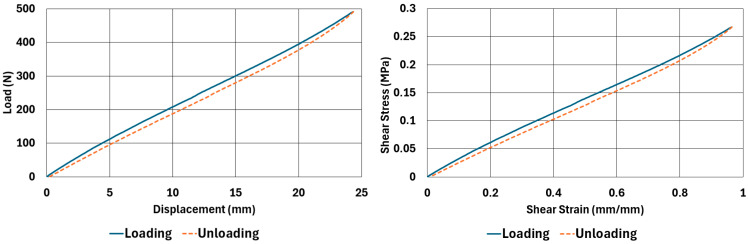
NR50 sample—shear test results at 0.033 1/s strain rate.

**Figure 9:**
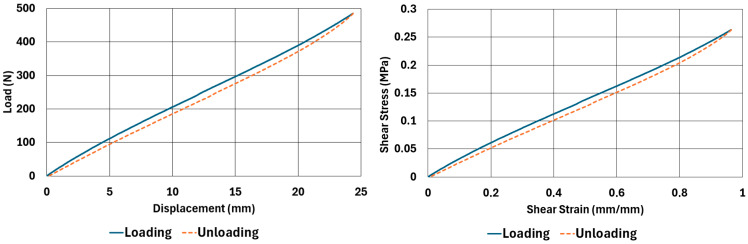
NR50 sample—shear test results at 0.013 1/s strain rate.

**Figure 10:**
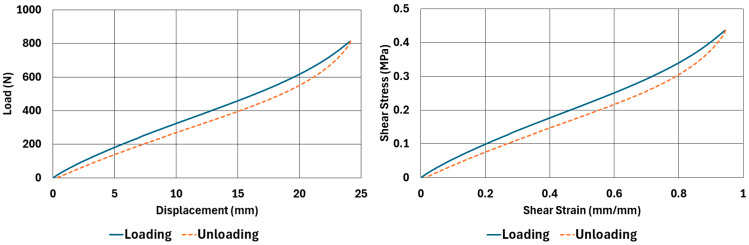
NR60 sample—shear test results at 0.033 1/s strain rate.

**Figure 11:**
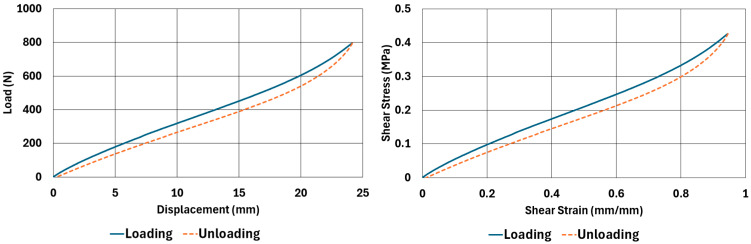
NR60 sample—shear test results at 0.013 1/s strain rate.

Using each sample’s geometrical dimensions, load and displacement values were converted to stress and strain pairs. For compressive loading, true stress and true strain were used, but for shear loadings, engineering stress and strain formulations were used to convert each set of load–displacement data to stress–strain data. These results are also given in Figure 4, Figure 5, Figure 6, Figure 7, Figure 8, Figure 9, Figure 10 and Figure 11 for each corresponding test sample. Also, for each set of data, a hysteresis value is calculated using Equation (1). The results are tabulated in Table 6. One can conclude that the hysteresis values are much higher when the samples are loaded under compression than shear. Also, the NR60 compound samples have much higher hysteresis values compared to the ones of the NR50 samples. That means that using a higher ratio of carbon black to base elastomer increases the hysteresis value of the elastomers.

(1) $$HYS=\left(\frac{E_{in}-E_{out}}{E_{in}}\right)\times 100=\frac{E_{loss}}{E_{in}}\times 100$$

**Figure 12:**
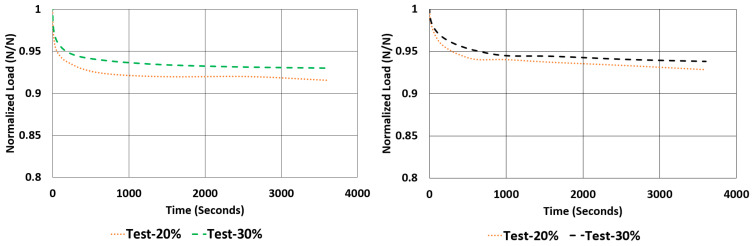
NR50 samples—relaxation test results under compression and shear.

**Figure 13:**
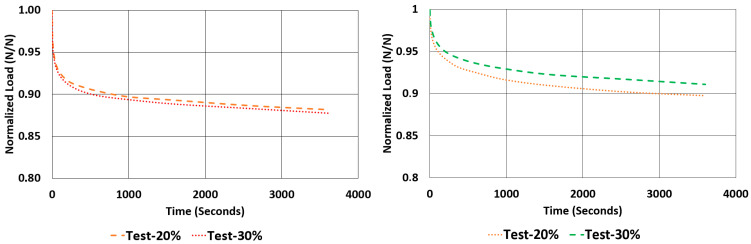
NR60 samples—relaxation test results under compression and shear.

Another important property to look for in a rubber component is the relaxation rate. For each sample, the relaxation rates are calculated and tabulated in Table 7 using the data given in Figure 12 and Figure 13. This value is approximated by finding the difference between the normalized values of loads at 1000 s and 3600 s and dividing it by 2600 s (as given in Equation (2)).

(2) $$Rate\;of\;Relaxation\;\left(ROR\right)=\frac{\Delta F}{\Delta t}=\frac{F_{3600}-F_{1000}}{2600}$$

The ROR values showed a negative relationship when they are compared to the value of compound hardness. As the hardness increases, the value of ROR decreases, resulting in higher changes in the normalized load value at each mean strain value in relaxation tests. Surprisingly, the value of ROR is always higher when under shear loads than compressive loads for the same rubber compound although the hysteresis values calculated for shear samples were almost half the hysteresis values calculated for compression samples.

When looking at the relaxation curves given in Figure 12 and Figure 13, we can obviously see a trend between the time interval 1000 s and 3600 s. So, these curves can be used to have an idea about the time-dependent viscoelastic behaviour of each material under compression and shear loadings.

## 4. Theory

As it was explained and summarized in Section 1, both hyperelastic and viscoelastic mechanical properties of elastomer materials are always at the interest of researchers because these materials are widely applied to many engineering solutions to sort out vibration, control, and noise problems.

Elastomers and elastomer-like materials exhibit dramatical changes in their mechanical properties in time and under cyclic loads applied. Also, the value of temperature is also an important property on the change in mechanical properties of elastomers. The change in the mechanical properties of elastomers is said to be viscoelastic properties and should modelled for applications where the changes in mechanical properties of components due to time, frequency, and other degraders are important.

**Figure 14:**
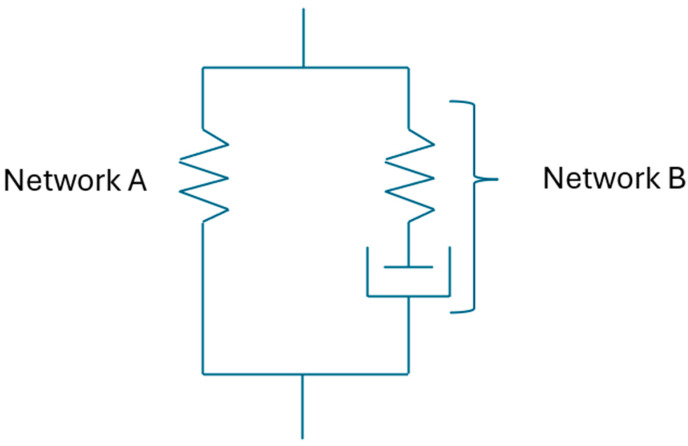
Network decomposition of mechanical responses of elastomers.

This study assumes that the mechanical properties of elastomer materials can be defined using two networks: Network A and Network B. Network A is the time-independent equilibrium response and Network B is the time-dependent response of elastomer materials as shown in Figure 14. These network chains are in parallel to each other [9].

In this study, Arruda–Boyce (Network A) and Bergström–Boyce (Network B) models are used to predict the viscoelastic response of rubber material under specific loading conditions. So, it is important to understand the theory behind it. Arruda–Boyce model is used as the main constitutive hyperelastic material model to define the time-independent response. It can also be called the equilibrium response. This model uses the following strain energy density Equation [8].

(3) $$W=\mu \sum_{i=1}^{5}\frac{C_{i}}{\lambda_{m}^{2i-2}}\left({\bar{I}}_{1}^{i}-3^{i}\right)$$

In Equation (3), μ is initial shear modulus, $\lambda_{m}$ is locking stretch (maximum stretch before chains become fully extended), ${\bar{I}}_{1}$ is first invariant of the deviatoric part of the right Cauchy–Green deformation tensor, $C_{i}$ are coefficients derived from inverse Langevin function series expansion. These coefficients are $C_{1}=1/2$, $C_{2}=1/20$, $C_{3}=11/1050$, $C_{4}=19/7000$, and $C_{5}=519/673750$.

To have a better curve fit, the values of μ and $\lambda_{m}$ may be final tuned after having statistical analysis of the curve-fit function in comparison to the real material test data.

To model the non-equilibrium response of the material, the Bergström–Boyce model is used in conjunction with the equilibrium response. The time- and rate-dependent viscoelastic material model can be expressed by the following Equation [9].

(4) $${\dot{F}}_{\vartheta }=D_{\vartheta }F_{\vartheta }$$

where $F_{\vartheta }$ is the deformation gradient of the viscoelastic network and $D_{\vartheta }$ is the viscous deformation rate. The viscous shear rate can be expressed as follows.

Now, one can define the strain energy function by using the following equation.

(5) $$\varphi \left(\bar{C},{\bar{C}}_{e}\right)=U\left(J\right)+W\left(\bar{C}\right)+W_{v}\left({\bar{C}}_{e}\right)$$

In this equation, $U\left(J\right)$ and $W\left(\bar{C}\right)$ are the strain energy functions in the time-independent network. These functions represent volumetric and volume-preserving parts, respectively. $W_{v}\left({\bar{C}}_{e}\right)$ is the volume-preserving strain energy due to viscous contribution, J is the determinant of deformation gradient, $\bar{C}$ is the volume-preserving part of total right Cauchy–Green deformation tensor, and ${\bar{C}}_{e}$ is the elastic volume-preserving part or right Cauchy–Green deformation tensor from viscous contribution. Bergström–Boyce defined the viscoelastic strain as

(6) $$\dot{\gamma }={\dot{\gamma }}_{0}\cdot {\left[\lambda_{chain}^{i}-1\right]}^{C_{2}}{\left(\left\|\frac{\tau_{iso}^{v}/\sqrt{2}}{\tilde{\tau }}\right\|\right)}^{m}$$

where $\lambda_{chain}^{i}$ is the inelastic network stretch, and $\tau_{iso}^{v}$ is the deviatoric Kirchhoff stress from viscous contribution.

At Marc 2024.2 the value of $\tilde{\tau }$ is the normalization value having the same units of stress. The effective creep strain rate then is defined by the following equation.

(7) $$\dot{\gamma }=C_{1}\cdot {\left[\lambda_{chain}^{i}-1\right]}^{C_{2}}{\left\|\tau_{iso}^{v}\right\|}^{m}$$

Here in this equation, $C_{1}$, $C_{2}$, and m are material parameters and can be curve-fitted using experimental stress–strain data at two different strain-rate values. Equation (7) can be reformed to have the following form.

(8) $${\dot{\Upsilon }}_{\vartheta }={\dot{\Upsilon }}_{0}{\left(\frac{\tau_{\vartheta }}{\tau_{0}}\right)}^{m}$$

where ${\dot{\Upsilon }}_{\vartheta }$ is viscous shear rate, $\tau_{\vartheta }$ is driving stress in non-equilibrium network, ${\dot{\Upsilon }}_{0}$ is rate sensitivity, $\tau_{0}$ is flow resistance, and m is the rate-sensitivity exponent. All these parameters can be fine-tuned to represent as accurate as possible viscoelastic material response.

In this study, the coefficients needed to define Arruda–Boyce and Bergström–Boyce models were obtained using the experimental data fit tools given in Marc 2024.2 software. The procedure for this is first to input the stress and strain data obtained from quasi-static tests to obtain the coefficients needed to define the time-independent response (Arruda–Boyce model) of the material (Network A). The stress–strain data at this stage should be a single set of data. Ascending and descending stress–strain data can be used to obtain the average curve that defines the time-independent equilibrium response of the material (this study suggests using the stress–strain data measured at lower strain rate to find the coefficients of the Arruda–Boyce model). In the second stage, ascending and descending stress–strain values obtained at two different strain-rate values are input to the experiment data fit tool already available to calculate the Bergström–Boyce model coefficients. For both computational curve-fit calculations a relative error tolerance of 1 × 10^−7^ was achieved.

## 5. Analysis Setup and Boundary Conditions

A good viscoelastic material model, which reflects the real physical behaviour of elastomer materials studied in this work, is a very essential part to describe the real physical behaviour of components made from the same materials. In addition to the definition of material models, the definition of boundary conditions also plays a very important factor to have a fair foundation to compare the results of finite element calculations with the results of real tests. So, in this section, detailed explanations are given to describe how the material model coefficients are calculated, and boundary conditions are defined for each loading condition and material.

To run the transient analyses, Marc Mentat 2024.2 was used. Also, to find the material model coefficients, the curve-fitting tools given in the same software were used. In Table 8, the coefficients used to characterize the material models are given. These coefficients were obtained using the curve fitting tools given in Marc Mentat 2024.2 software. These tools can calculate both Arruda–Boyce and Bergström–Boyce material coefficients for a given set of stress–strain data in various loading modes. In this study, uniaxial compression and simple shear stress–strain data (data shown between Figure 3, Figure 4, Figure 5, Figure 6, Figure 7, Figure 8, Figure 9 and Figure 10) are given to calculate the corresponding coefficients as given in Table 8.

The FEM models were constructed such that the computation time is minimized. Therefore, for compression and shear samples, 2D axisymmetric and 2D plain strain formulations were chosen for the elements, respectively.

**Figure 15:**
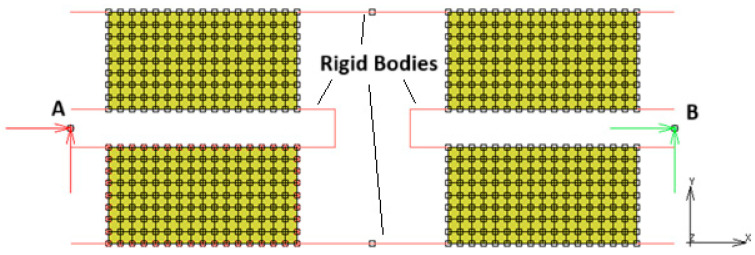
FEM model of simple shear sample.

In Figure 15, the FEM model of the shear sample is given. This model uses 512 four-noded quad elements and 612 nodes. The elements use “plain strain with full & Herrmann formulation”. The upper and lower lines represent the floating metal parts, which connect the rubber pads in the element. These lines are not constrained in both X and Y directions, but they create rigid links between the adjacent rubber blocks (no contact was used). The node highlighted by letter A is fully fixed in X and Y directions, while the node highlighted by letter B is used to apply displacement boundary conditions in the time domain.

**Figure 16:**
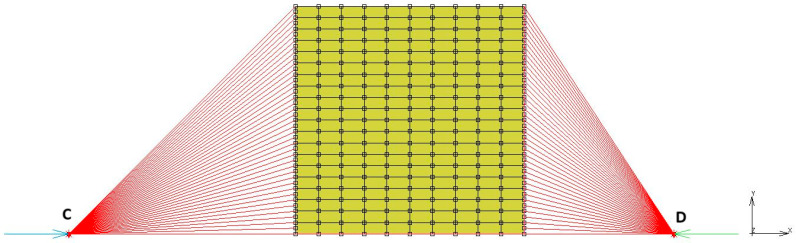
FEM model of compression sample.

The FEM model shown in Figure 16 uses 200 four-noded quad elements and 231 nodes. The elements use axisymmetric full and Herrmann formulation. The axisymmetric axis coincides with the absolute “x” axis shown in Figure 13. Two RBE2 links were used to represent the surfaces, which were used to compress the rubber discs in between. To conduct this without using rigid lines, relative nodes defined in RBE2 links are not constrained in Y direction. The node highlighted by letter C was used to fix the whole model, while the node highlighted by letter D was used to define displacement boundary conditions in the time domain. The same test conditions given in Table 4 and Table 5 were used as the initial and boundary conditions to run the corresponding analyses.

The elements (Type 82) used in this study are of four-node, iso-parametric quadrilateral elements suitable to use for incompressible applications. The displacement formulation of these elements is modified using the Hermann variational principle. A Pardiso Direct Sparce solver was used to solve every FEM problem. The relative force convergence ratio has been set to 0.5 all runs to test the convergence of computations. No friction model was used in the analyses, because friction was assumed to have a neglectable effect.

## 6. Analysis Results

**Figure 17:**
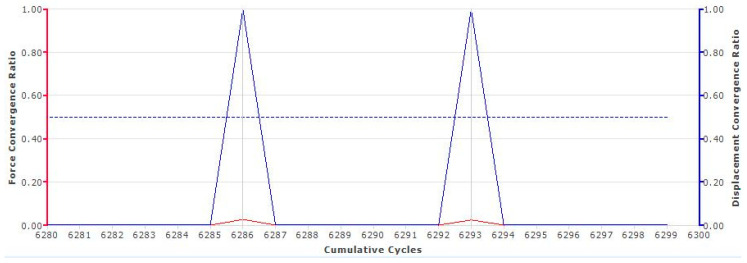
Force and displacement convergence ratio vs. cumulative cycles graph for one of the runs.

As it was earlier mentioned, the relative force convergence criteria were taken as 0.5. For the complete study, 16 different transient analyses were run. Below a convergence ratio graph throughout the course of iterative cycles is given for NR50 Compression sample. As it can be seen from Figure 17, the analysis error does not increase above 0.1 throughout the complete run. This has been always the case for all 16 different transient analyses carried out in this study.

**Figure 18:**
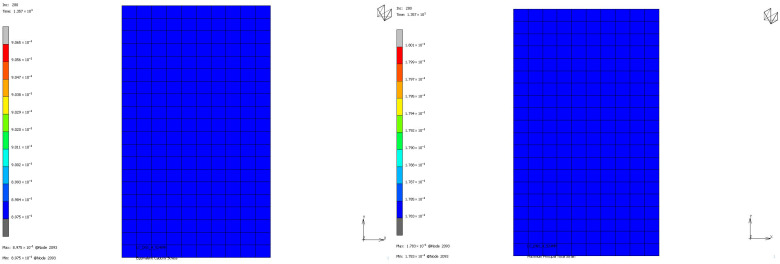
Equivalent Cauchy stress and maximum principal total strain graph at ε = −0.3 mm/mm strain for NR50 compression sample.

**Figure 19:**
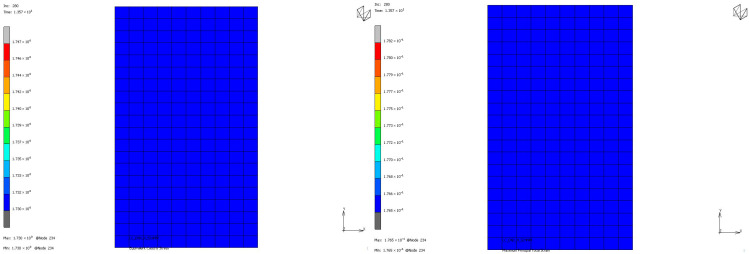
Equivalent Cauchy stress and maximum principal total strain graph at ε = −0.3 mm/mm strain for NR60 compression sample.

**Figure 20:**
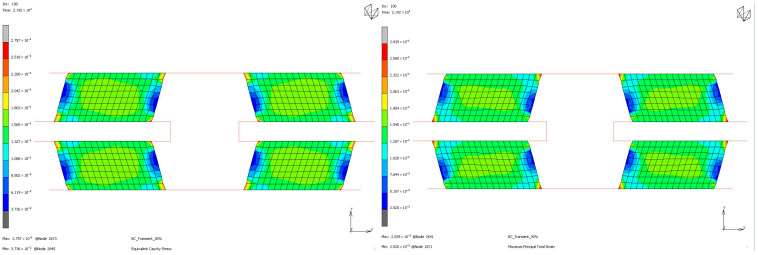
Equivalent Cauchy stress and maximum principal total strain graph at ϑ = −0.3 mm/mm strain for NR50 shear sample.

**Figure 21:**
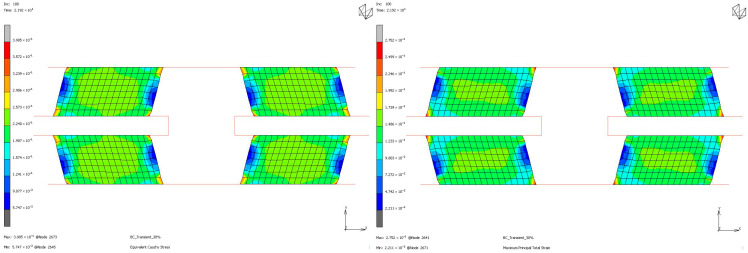
Equivalent Cauchy stress and maximum principal total strain graph at ϑ = −0.3 mm/mm strain for NR60 shear sample.

To compare the mechanical properties of materials using stress and strain distributions at the deformation state where the nominal value of engineering strain reaches 0.3 for all loading modes, contour plots are given for all samples to see the distribution in both the value of equivalent Cauchy stress and the value of maximum principal total strain in Figure 18, Figure 19, Figure 20 and Figure 21. The first observation is that for all stress and strain values computed under compressive loadings, the quantitative values are all the same and equal. This is because the finite element model setup has no contact definition and the load applied is compressing the elements in only the X direction homogeneously at every increment of analysis. If one would like to compare the results under compressive loads, it can be said that the value of stress is almost doubled for the NR60 sample compared to the same value of NR50. In other words, at the same deformation state the reaction force obtained from the NR60 sample will be much higher than the reaction force obtained from the NR50 sample. In this case these values are 906 N for the NR50 sample and 1756 N for the NR60 sample. For shearing loads, the values of equivalent Cauchy stress are 0.28 MPa and 0.39 MPa for the NR50 and NR60 shear samples, respectively. The resulting reaction forces are 50 N and 77 N for the NR50 and NR60 shear samples, respectively. Using a higher hardness compound is way more effective to obtain reaction loads relatively much higher is only possible when the loading mode is under compression.

**Figure 22:**
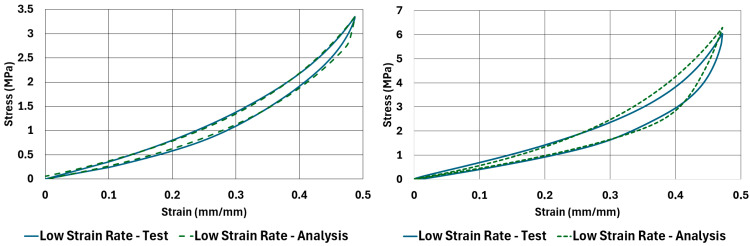
Test versus analysis results for compression samples of NR50 and NR60 materials at low strain-rate values (ANOVA *p*-values 0.865822 and 0.104927 for left and right graphs, respectively).

**Figure 23:**
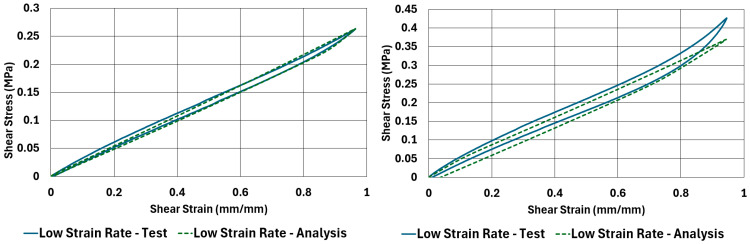
Test versus analysis results for shear samples of NR50 and NR60 materials at low strain-rate values (ANOVA *p*-values 0.733354 and 0.143516 for left and right graphs, respectively).

Graphical comparison between test results and analysis results of the stress–strain curves of the NR50 and NR60 samples under compressive and shear loadings are given in Figure 22 and Figure 23. These graphs show very good agreement between the real test results and computation results.

The comparison results of compression samples can be compared to the same comparison made by Bergström and Boyce [9]. In their study, there was no sample tested under shear loadings. The material was also different. They used a chloroprene-based rubber compound with various carbon black ratios. One of the carbon black ratio they used was 65 phr, which is comparable to the NR60 compound formulation used in this study. According to the plots shown by Bergström and Boyce [9], the maximum true stress value is around 3.5 MPa at around 0.58 mm/mm true strain value. To compare the same values for the results of the NR60 compression sample, the maximum engineering stress values need to be converted to true stress–strain values. An engineering stress value of 6.29 MPa can be converted to 3.33 MPa to find the equivalent true stress value. The engineering strain value can also be calculated as 0.65 mm/mm. Considering the lower content of carbon black present in NR60 formulation, these values are considerably comparable to each other.

Another good way to compare the results is to compare the corresponding hysteresis value of each analysis result with respect to the test results. This comparison is given in Table 9. Considering the deviations reported in this table, the computation results give reliably comparable values compared to real test values.

In theory, viscoelastic mechanical properties like creep and relaxation are highly correlated with hysteresis. There is an inverse relationship between them. Therefore, it is worth examining this relationship between results (both numerical and real test results). A considerably fair estimation of relaxation behaviour of elastomeric products using only hysteresis data can really speed up development of such components for industrial purposes. This comparison is given in Table 10, Table 11, Table 12 and Table 13.

Hysteresis results obtained from tests and computations have shown very good agreement, such that the maximum deviation between the results is only −2.2%. The curves are also very similar to each other graphically. That means these models can be used to predict load–deformation relationships of components using the materials investigated in this study.

The rate of relaxations (RORs) obtained in tests and computations have shown obvious differences. However, normalized load values at the end of relaxation tests and computations are very close to each other. The maximum deviations encountered for these values are not more than −3.33% and 1.07%. Considering the total drop in the total normalized relaxation values obtained using tests and computations are giving a relationship, which was expected from theoretical point of view. For example, in Table 13, for the NR50 compression case, computations gave a total drop of 0.08 while the real test values gave a total drop of 0.07. There is a deviation of 14.3%. In the case of NR50 shear, these values are obtained as 0.06 and 0.05 from test and computation, respectively. This corresponds to a deviation of 16.7% for this case. When the same comparison is made for the NR60 compression case, the corresponding relaxation drop values are 0.13 and 0.15, calculated from test and computation, respectively. For this specific case the total deviation calculated is 15.4%. Finally, for the last case of NR60 shear relaxation, the total drops of test and computation are both 0.09 and have no deviation. This is another good indication that the modelling procedure recommended in this paper is a considerably good alternative to have an idea about the time-dependent viscoelastic mechanical property change in a component made from elastomer or elastomer-like material.

**Figure 24:**
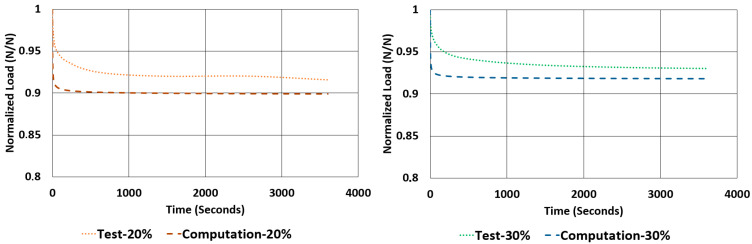
Graphical comparison of normalized relaxation results between tests and computations under compressive strains of 20% and 30% for NR50 material (ANOVA *p*-values 0.999918 and 0.999936 for left and right graphs, respectively).

**Figure 25:**
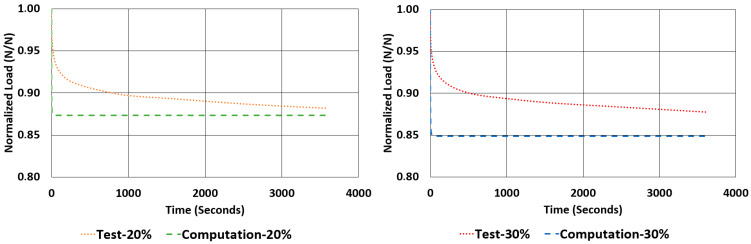
Graphical comparison of normalized relaxation results between tests and computations under compressive strains of 20% and 30% for NR60 material (ANOVA *p*-values 0.999878 and 0.999846 for left and right graphs, respectively).

**Figure 26:**
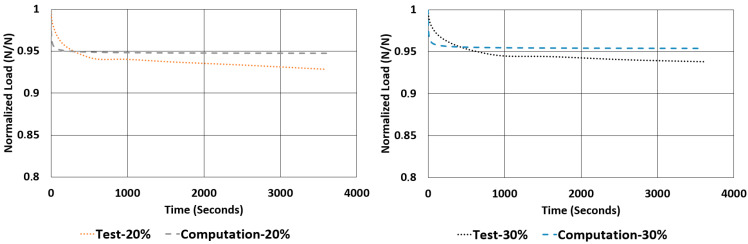
Graphical comparison of normalized relaxation results between tests and computations under shear strains of 20% and 30% for NR50 material (ANOVA *p*-values 0.999982 and 0.999985 for left and right graphs, respectively).

**Figure 27:**
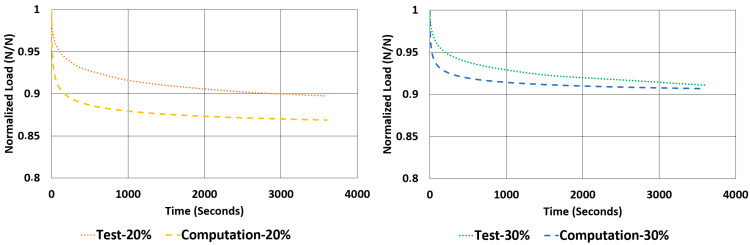
Graphical comparison of normalized relaxation results between tests and computations under shear strains of 20% and 30% for NR60 material (ANOVA *p*-values 0.999927 and 0.999961 for left and right graphs, respectively).

Considering Figure 24, Figure 25, Figure 26 and Figure 27, generally, relaxation behaviour computed using the FEM model is converging the real test values as the test duration is increased. To have a better understanding about the curves given in Figure 22, Figure 23, Figure 24, Figure 25, Figure 26 and Figure 27, an ANOVA two-factor with replication statistical analysis was carried out to find how much the curves are like each other. The corresponding *p*-values are given for each graph in each corresponding caption of Figure 22, Figure 23, Figure 24, Figure 25, Figure 26 and Figure 27. These values were calculated above 0.05 for all cases considered. Further studies can be carried out testing the samples against relaxation in longer durations.

## 7. Conclusions

Considering the results presented in this paper, a reliable time-dependent relaxation analysis can be conducted using the Bergström–Boyce model obtained from simple hysteresis tests. Although the rate values do not converge to each other, the viscoelastic models given in this paper can be used to estimate the total relaxation (in a longer time domain) of any component made from a material discussed in this study. The analysis results are comparable to the real test results within a deviation of +1.07% to −3.33% for the samples analyzed within the scope of this study. This approach can be used for components whose viscoelastic properties are needed to be known for relatively longer time periods, such as seismic isolators, bridge flexible joints, suspension elements working under relatively very high loads, and sealing elements needing relatively very long-life spans.

## Figures and Tables

**Table 1:** Curing parameters of samples.

Material	Sample Type	Temperature (°C)	Duration (Minutes)	Pressure (Bar)
NR50	Compression	150	8	200
	Simple Shear	150	9	200
NR60	Compression	150	8	200
	Simple Shear	150	7	200

**Table 2:** Geometrical properties of compression samples.

Sample	Diameter (mm)	Height (mm)
NR50	30.00	15.08
NR60	30.13	15.11

**Table 3:** Geometrical properties of simple shear samples.

Sample	Height (mm)	Width (mm)	Depth (mm)
NR50	12.62	12.32	24.94
NR60	12.77	12.41	25.03

**Table 4:** Test parameters of compression samples.

Preload (N)	−10
Number of Preconditioning Cycles	3
Number of Test Cycles	1
Strain Rate, $\dot{\varepsilon }$ (1/s)	−0.0221/−0.0553
Test Limits, ε (mm/mm)	0–(−0.5)

**Table 5:** Test parameters of simple shear samples.

Preload (N)	10
Number of Preconditioning Cycles	3
Number of Test Cycles	1
Strain Rate, $\dot{\vartheta }$ (1/s)	0.013/0.033
Test Limits, ϑ (mm/mm)	0–1

**Table 6:** Hysteresis (HYS) values calculated for each sample in each loading mode (the maximum strain limits of tests are ≈0.5 and ≈1 for compression and shear, respectively.

Sample	Loading Mode	HYS (%)
NR50	Compression	16.45
NR50	Shear	7.06
NR60	Compression	26.41
NR60	Shear	13.24

**Table 7:** ROR calculated for each sample in each loading mode.

Sample	Loading Mode	Mean Strain (%)	ROR (1/s)
NR50	Compression	20	−2.23 × 10^−6^
NR50	Compression	30	−2.52 × 10^−6^
NR50	Shear	20	−4.55 × 10^−6^
NR50	Shear	30	−2.62 × 10^−6^
NR60	Compression	20	−5.88 × 10^−6^
NR60	Compression	30	−6.19 × 10^−6^
NR60	Shear	20	−7.17 × 10^−6^
NR60	Shear	30	−7.04 × 10^−6^

**Table 8:** Material coefficients used to model the materials.

Material	Loading Mode	Arruda–Boyce		Bergström–Boyce				
		μ (MPa)	$\lambda_{m}$ (−)	μ (MPa)	$\lambda_{m}$ (−)	${\dot{\Upsilon }}_{0}$	$\tau_{0}$	m
NR50	Compression	0.775312	6.19388	1.61734	1.37164	154.145	−0.70877	3.7
NR50	Shear	0.282883	66.6866	0.138175	2.56569	1143.25	−1	3.108
NR60	Compression	1.21051	3.3307	2.34232	3.3307	0.0185289	−1	1.5
NR60	Shear	0.399043	39.9397	0.101834	2.47915	1.547 × 10^7^	0	5.57

**Table 9:** Comparison of hysteresis values calculated by test results and analysis results.

Sample	Loading Mode	Hysteresis—Test (%)	Hysteresis—Analysis (%)	Deviation (%)
NR50	Compression	16.45	16.08	−2.2
NR50	Shear	7.06	7.07	0.14
NR60	Compression	26.41	26.6	0.72
NR60	Shear	13.24	13.25	0.08

**Table 10:** Correlation between hysteresis and ROR at 20% constant strain.

Sample	Loading Mode	Hysteresis Test (%)	Hysteresis Analysis (%)	Deviation (%)	Relaxation Rate @Test (1/s)	Relaxation Rate @Analysis (1/s)
NR50	Comp.	16.45	16.08	−2.2	−2.23 × 10^−6^	−3.83 × 10^−7^
NR50	Shear	7.06	7.07	0.14	−4.55 × 10^−6^	−3.08 × 10^−7^
NR60	Comp.	26.41	26.6	0.72	−5.88 × 10^−6^	0
NR60	Shear	13.24	13.25	0.08	−7.17 × 10^−6^	4.09 × 10^−6^

**Table 11:** Correlation between hysteresis and ROR at 30% constant strain.

Sample	Loading Mode	Hysteresis Test (%)	Hysteresis Analysis (%)	Deviation (%)	Relaxation Rate @Test (1/s)	Relaxation Rate @Analysis (1/s)
NR50	Comp.	16.45	16.08	−2.2	−2.52 × 10^−6^	−4.81 × 10^−7^
NR50	Shear	7.06	7.07	0.14	−2.62 × 10^−6^	−3.10 × 10^−7^
NR60	Comp.	26.41	26.6	0.72	−6.19 × 10^−6^	0
NR60	Shear	13.24	13.25	0.08	−7.04 × 10^−6^	−2.87 × 10^−6^

**Table 12:** Correlation between hysteresis and total normalized relaxation at 20% constant strain.

Sample	Loading Mode	Hysteresis Test (%)	Hysteresis Analysis (%)	Deviation (%)	Total Normalized Relaxation@ Test (−)	Total Normalized Relaxation@ Analysis (−)	Deviation (%)
NR50	Comp.	16.45	16.08	−2.2	0.92	0.90	−2.17
NR50	Shear	7.06	7.07	0.14	0.93	0.94	1.07
NR60	Comp.	26.41	26.6	0.72	0.88	0.87	−1.13
NR60	Shear	13.24	13.25	0.08	0.90	0.87	−3.33

**Table 13:** Correlation between hysteresis and total normalized relaxation at 30% constant strain.

Sample	Loading Mode	Hysteresis Test (%)	Hysteresis Analysis (%)	Deviation (%)	Total Normalized Relaxation@ Test (−)	Total Normalized Relaxation@ Analysis (−)	Deviation (%)
NR50	Comp.	16.45	16.08	−2.2	0.93	0.92	−1.08
NR50	Shear	7.06	7.07	0.14	0.94	0.95	1.06
NR60	Comp.	26.41	26.6	0.72	0.87	0.85	−2.30
NR60	Shear	13.24	13.25	0.08	0.91	0.91	0

## Data Availability

The original contributions presented in the study are included in the article, further inquiries can be directed to the corresponding author.

## Abbreviations

The following abbreviations are used in this manuscript:

HYS	Hysteresis
ROR	Rate of relaxation
FEM	Finite element method
NR	Natural rubber
CB	Carbon black
BR	Butadiene rubber
SBR	Styrene butadiene rubber
XIIR	Halogenated polyisobutylene rubber
Phr	Per hundred rubber
ShA	Shore A
RBE2	Rigid body element 2
SVR	Standard Vietnamese rubber
CV	Constant viscosity
FEF N	Fast extrusion furnace Normal
IPPD	N-Isopropyl-N′-Phenyl-p-Phenylenediamine
TMQ	2,2,4-Trimethyl-1,2-Dihydroquinoline
TBZTD	Tetrabenzylthiuram Disulfide
TBBS	N-tert-Butyl-2-benzothiazolesulfenamide
S	Sulphur
RPA	Rubber process analyzer
2D	Two dimensional
MPa	Mega pascal
F	Force
